# Protein Conformation Governs Spin-Selective Electron Transmission

**DOI:** 10.1021/acs.jpclett.5c01495

**Published:** 2025-06-17

**Authors:** Naupada Preeyanka, Tapan Kumar Das, Ron Naaman

**Affiliations:** Department of Chemical and Biological Physics, 34976 Weizmann Institute of Science, Rehovot 7610001, Israel

## Abstract

The chiral induced spin selectivity (CISS) effect results in spin-dependent electron transmission through chiral systems. In biological systems such as proteins, chirality appears in both primary and secondary structures, namely, in the existence of asymmetric carbon atoms and in the chiral configuration of oligopeptide subunits. An important question is what contribution each type of chirality makes to this effect. Here we present the impact of denaturation on spin polarization using d-glucose oxidase (GOx) as a model system. Employing Hall-effect and magnetoresistance (MR) measurements, we compared the spin selective behavior of GOx in its native and thermally denatured states. Our results show that the native protein, characterized by a well-defined helical structure and intact flavin adenine dinucleotide (FAD) cofactor, exhibits strong spin polarization. Upon denaturation at elevated temperatures (65 and 95 °C), a marked reduction in both Hall voltage slope and MR values indicates a significant loss in spin polarization capability. This behavior is attributed to the disruption of the protein’s secondary structure, which is essential for maintaining chiral potential landscapes for spin selectivity. These findings highlight the importance of secondary structure in maintaining a high spin polarization in proteins. We also demonstrate that the spin-related structural properties of the protein are retained, even when the protein is imbedded in a solid-state device.

## Graphical abstract

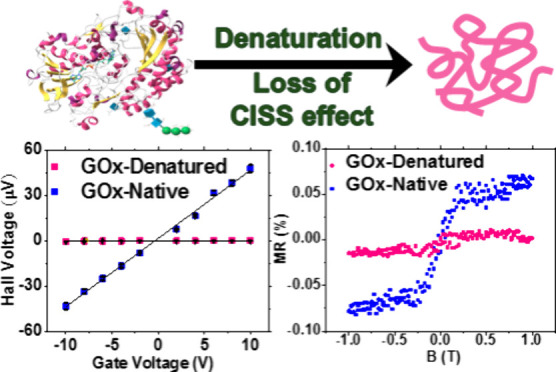

In the last two decades, many studies have revealed that electron transport through chiral biomolecules depends on the electrons’ spin. This phenomenon, known as chiral induced spin selectivity (CISS),^1^ has also been observed in proteins^2–6^ and in bacterial extracellular electron transfer processes.^7,8^ A recent study on the association between DNA and model primordial polypeptides^9^ demonstrated that spin polarization enhances the binding of peptides to DNA, making the docking^10^ of DNA on peptides more efficient. These studies indicate that chirality and electrons’ spin polarization may be important, and perhaps essential, properties in many biological processes.^11^

Chirality is present in proteins both in their primary structure—most amino acids are chiral—and in their secondary structure, particularly in α-helices. An important question arises: which type of chirality contributes more significantly to spin polarization?

The proper folding of a protein is governed by various stabilizing forces, including hydrogen bonds, disulfide bonds, and hydrophobic interactions.^12^ Disruptions to these interactions, whether due to changes in pH, temperature, or the presence of denaturing agents, can lead to structural alterations, collectively known as denaturation, ultimately affecting the protein’s biological function.^13^

In this study, we examined the dependence of spin polarization on the structure of native versus denatured d-glucose oxidase (D-GOx), a dimeric flavoprotein. The three-dimensional structure of GOx is primarily stabilized by its Flavin adenine dinucleotide (FAD) cofactor, which is essential for both its catalytic activity and proper assembly, as well as overall protein stability.^14^ To induce denaturation, we varied the temperature of a solution containing the protein within the range of 65–95 °C for 10 min, then adsorbed the protein on the substrate, and monitored the correlation between the protein structure and spin-dependent transport. Spin polarization was measured using Hall devices^15^ and magnetoresistance (MR) techniques.^16^

It is important to note that, in the past, the dependence of the CISS effect’s magnitude on secondary structure has primarily been studied in simple oligomers, such as double-stranded DNA^17^ and oligopeptides,^18^ where electron transmission occurs along essentially a single pathway. In proteins, however, their three-dimensional structure allows electrons to be directed or displaced through multiple pathways. Consequently, one could imagine that the impact of denaturation on the CISS effect may not be as dramatic as that in simpler systems. This study aims at verifying the secondary structure effect on the magnitude of the CISS effect in a full-size protein.

A schematic structure of GOx is shown in Figure 1a, where yellow arrows represent the β-sheets and magenta coils indicate the α-helices of the protein’s secondary structure. The length of GOx is ∼8–10 nm.^19^ The core functional unit of GOx is flavin adenine dinucleotide (FAD) (Figure 1b), which plays a key role in establishing the electron transfer pathway. GOx was chosen for this study due to its well-known catalytic activity and its widespread application as a model enzymatic system.^20,21^ It is hypothesized here that during protein unfolding and refolding, both the secondary structure and the FAD cofactor are involved in the structural transitions and that these events exhibit spin selectivity. To investigate this, the secondary structure of GOx was analyzed before and after denaturation using circular dichroism (CD) and polarization modulation infrared reflection absorption spectroscopy (PMIRRAS).

**Figure 1:**
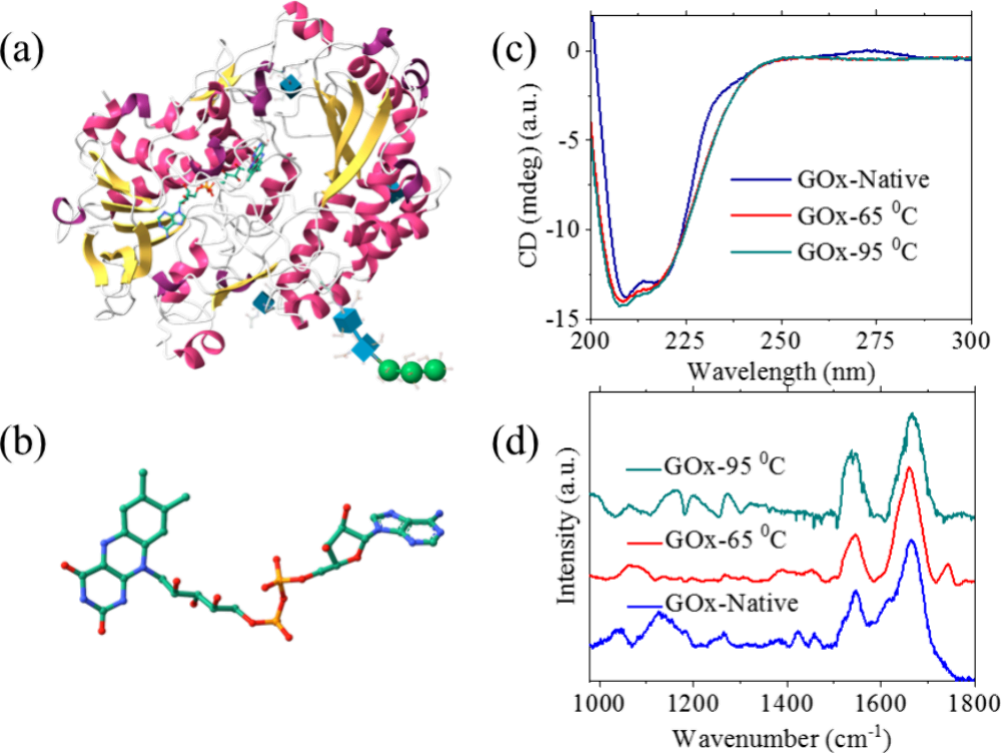
(a) Cartoon representation of d-glucose oxidase (GOx) showing the secondary structure comprising of β-sheets (yellow) and α-helices (magenta). (b) The molecular structure of FAD unit in GOx responsible for the enzymatic actions. (c) The CD spectra of native and denatured GOx (at 65 and 95 °C). (d) The polarization modulation infrared reflection absorption spectra (PMIRRAS) of native GOx along the denatured protein (65 and 95 °C).

CD spectroscopy is a valuable tool for detecting conformational changes in the active site of GOx because of the interaction between the flavin and tryptophan residues. Figure 1c shows the CD spectra of GOx in its native state and denatured state at 65 and 95 °C, respectively. The characteristic peaks at 208 and 222 nm in the far-UV region arise primarily from the *n* → π* transition associated with the protein secondary structure, specifically α-helices and β-sheets along with contributions from the aromatic side chain. The denaturation process can be monitored by following the ellipticity at 222 nm. Upon heating GOx to 65 and 95 °C and then cooling back to room temperature, the peak at 222 nm undergoes broadening and blue shifting, indicating partial or complete unfolding of the secondary structure, consistent with a denatured state. Moreover, the overall spectra become broader compared to those of the native state, supporting structural disruption. Interestingly, the persistence of the 222 nm peak even after heating suggests that partial refolding or recovery of the secondary structure occurs upon cooling. The band observed at 275 nm originates from coupling between the FAD cofactor and tryptophan residues in the active site.^22^ After denaturation, this peak disappears, indicating either the dissociation of the FAD cofactor or the disruption of its interaction with the protein matrix. To further prove these changes, PMIRRAS measurements were performed on D-GOx in both its native and its denatured states. Figure 1d presents the PMIRRAS spectrum of GOx in its native state and denatured at 65 and 95 °C. Figure S1 shows the PMIRRAS spectra of the individual denatured states of GOx. The peak at 1420 cm^–1^ corresponds to the in-plane bending vibration of the NH_2_ group in the FAD unit.^23^ A reduction in his peak intensity is observed after heating to 65 °C, while the band disappears completely at 95 °C. This suggests that the FAD either dissociates from the protein or its coupling with tryptophan residues is disrupted. The peaks at 1540 and 1650 cm^–1^ correspond to the amide II and amide I bands, respectively.^19,24^ The shoulder peak (1650 cm^–1^) which is highly sensitive to structural modifications can be seen in the native state of GOx but disappears during the denaturation process, while the peak at 1540 cm^–1^ is present even after denaturation. This indicates that although the protein undergoes unfolding, the amide backbone regions are at least partially retained, highlighting the resilience of certain structural elements.

To investigate the effect of the structure on the ability of the system to polarize moving electrons’ spins, we performed the Hall-voltage measurements using a GaN/AlGaN heterojunction device, which hosts a two-dimensional electron gas (2DEG) system (see Supporting Information).^14,25^ A schematic of the Hall device is shown in Figure 2a. The Hall channel, whose detailed composition is provided in Figure S2, is coated with a thin 5 nm gold layer to ensure effective immobilization of chiral molecules (linker + GOx) and to mitigate potential instability arising from surface charging effects in the semiconductor.^23,26^ An optical microscopy image of the device is shown in Figure S3. In the experiment, a self-assembled monolayer (SAM) of chiral GOx molecules was chemically adsorbed onto the Hall channel.^12^ The experiment was performed when the Hall device and the gate electrode are inserted in buffer solution, 0.1 M phosphate-buffer saline (PBS) at pH 7. A constant current was applied between the source (S) and drain (D) electrodes, while a gate voltage was applied perpendicular to the surface, in the absence of any external magnetic field. Because of the electrolyte solution, most of the potential falls between the adsorbed layer of the protein and the Hall device. The gate-induced electric potential facilitates charge displacement within the protein, leading to charge transfer between the substrate (Hall channel) and the adsorbed protein. In the case of negative gate potential, the system polarizes the electron’s spin; this charge displacement is at least partial spin polarized, due to the CISS effect, and hence electrons’ spins are injected into the Hall device and we measure a Hall voltage due to the anomalous Hall effect, occurring when electrons (conducted between the source and drain) are scattered from these spins.^27,28^ In the case of positive gate potential, spin polarized holes are injected into the Hall device. As was presented before, the exchange interaction between these injected spins is enhanced by the underlying 2DEG in the semiconductor substrate.^13^

**Figure 2:**
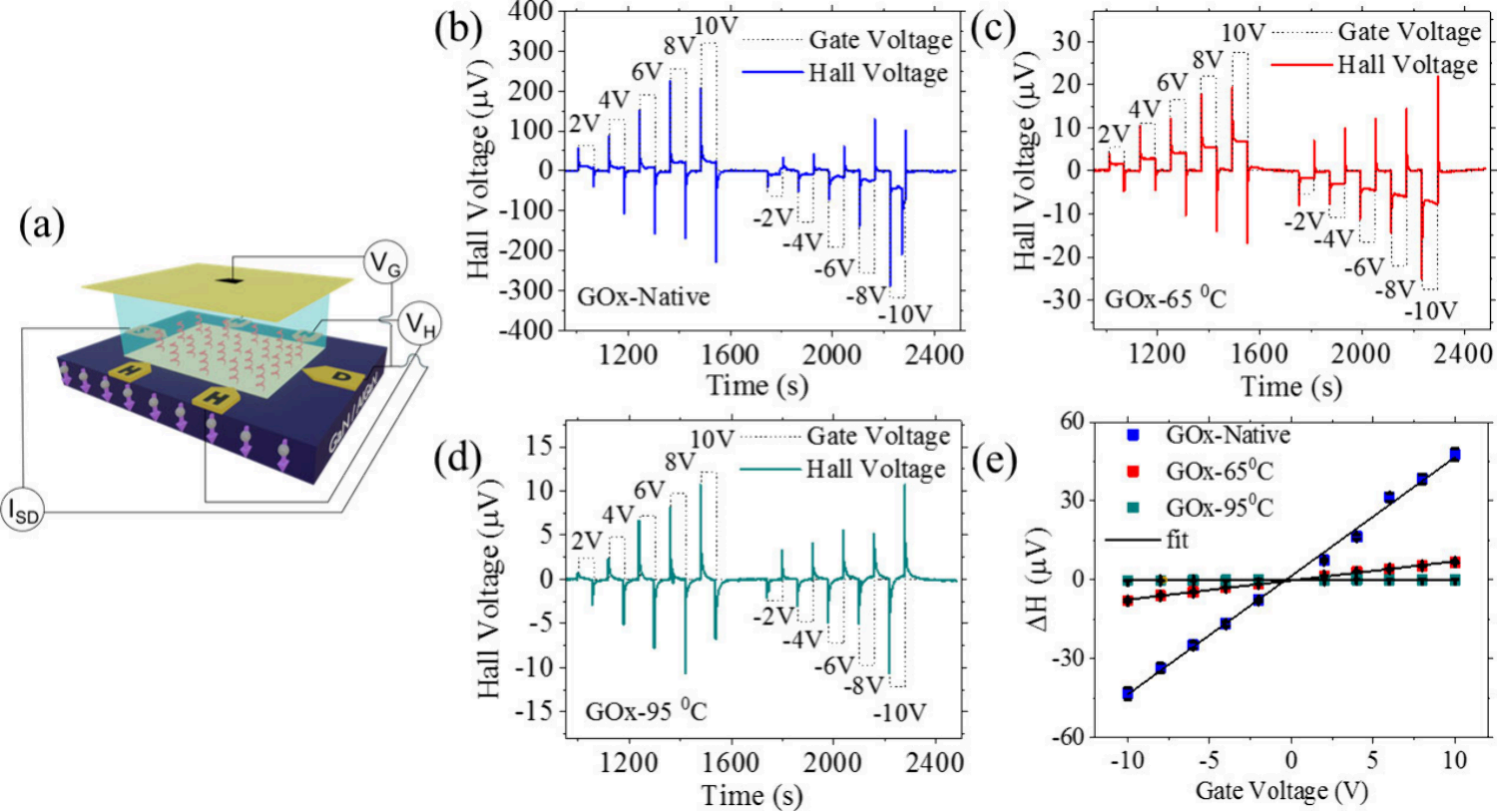
Hall device and results. (a) Schematic representation of the Hall device setup for spin-polarization studies, where the device is inserted in buffer solution and a gate electrode (yellow plane) is located at a distance of about 3 mm from the device. *V*_G_ is the gate voltage, *I*_SD_ is the current flowing between source and drain, and *V*_H_ is the Hall voltage between Hall probes. The device is imprinted on a GaN/AlGaN substrate with the protein (red colored spirals) adsorbed on the channel, which are connected to source (S) and drain (D) electrodes. (b) Hall results obtained for native, and for GOx denatured at 65 °C (c) and 95 °C (d). (e) The measured difference in Hall voltage (Δ*H*) as a function of gate voltage for GOx in native and denatured states. The sharp spikes in the signal are due to electrical noise resulting from turning on/off of the voltage. They are subtracted to obtain the actual Hall signal.

The Hall voltage signal was measured as a function of the gate voltage pulses applied to the protein adsorbed on the Hall device channel. The measurements were conducted at room temperature on the native proteins and in their denatured states. The Hall signal is shown in Figure 2b–d. A sequence of gate voltage pulses, from −10 V to +10 V with steps of 2 V, were applied across the channel and the gate. The Hall signal obtained from the native and denatured GOx as a function of applied gate voltage shows a transient peak appearing at each value of the gate voltage and then a fast decay. This decay is due to the formation of a double layer in the PBS solution. The exact method for extracting the Hall voltage can be found elsewhere.^14^ The difference in the Hall signals (Δ*H*) is linearly dependent on the applied gate voltage (Figure 2e). This linear dependence of the slope of the applied negative/positive potential is a consequence of the anomalous Hall effect. The slope of the Hall signal, as a function of the gate potential, is proportional to the spin polarization efficiency in the adsorbed protein. It is evident from these Hall measurements that upon denaturation, the spin polarization in the protein is reduced significantly.

To verify the Hall results, we investigated electron spin transport through denatured protein using a magnetoresistance (MR) device. A spin-valve device was used for this study in a crossbar geometry on a Si/SiO_2_ substrate.^14^ A self-assembled monolayer (SAM) of protein was adsorbed on the bottom electrode, which is made of a 5 nm titanium (Ti) adhesion layer followed by a 60 nm gold (Au) layer. A 1.5 nm magnesium oxide (MgO) layer was deposited as the blocking layer to avoid pinholes on the top of the protein SAM. A ferromagnetic nickel (Ni) layer was thermally evaporated on the MgO layer as the top electrode. MR measurements were performed as a function of an external magnetic field (*B*) up to ±1T perpendicular to the sample surface at different temperatures while passing a constant current of 10 mA through the multilayer structure (Au/GOx/MgO/Ni), with current flow aligned perpendicular to the sample surface. Figure 3a schematically shows the device architecture and the experimental setup. The magnified interface region is depicted in the inset. The MR values of native GOx at various measuring temperatures (100, 200, and 280 K) are shown in Figure 3b. The MR curves exhibit a distinct asymmetry at all temperatures. This asymmetric behavior is a hallmark of the chiral induced spin selectivity (CISS) effect, arising from the interplay between the spin polarization direction of the ferromagnetic electrode and the handedness of the chiral protein interface.^1^ The magnetoresistance was calculated using the equation MR(%) = $\frac{R\left(B\right)-R\left(0\right)}{R\left(0\right)}\times 100$ , where *R*(*B*) is the resistance under an applied field and *R*(0) is the resistance at zero magnetic field. Clearly, there is no significant temperature-dependent effect on the MR values. Please note that all temperatures are below room temperature. To probe the effect of denaturation on the spin polarization, MR measurements were performed using both native protein and denatured protein (Figure 3c). Although the nature of the MR curve remains the same, the MR values reduce with the increase in denaturation (see Figure 3d). This decrease in spin-polarization, i.e., weak CISS effect, in the denatured proteins is consistent with the Hall-voltage measurements.

**Figure 3:**
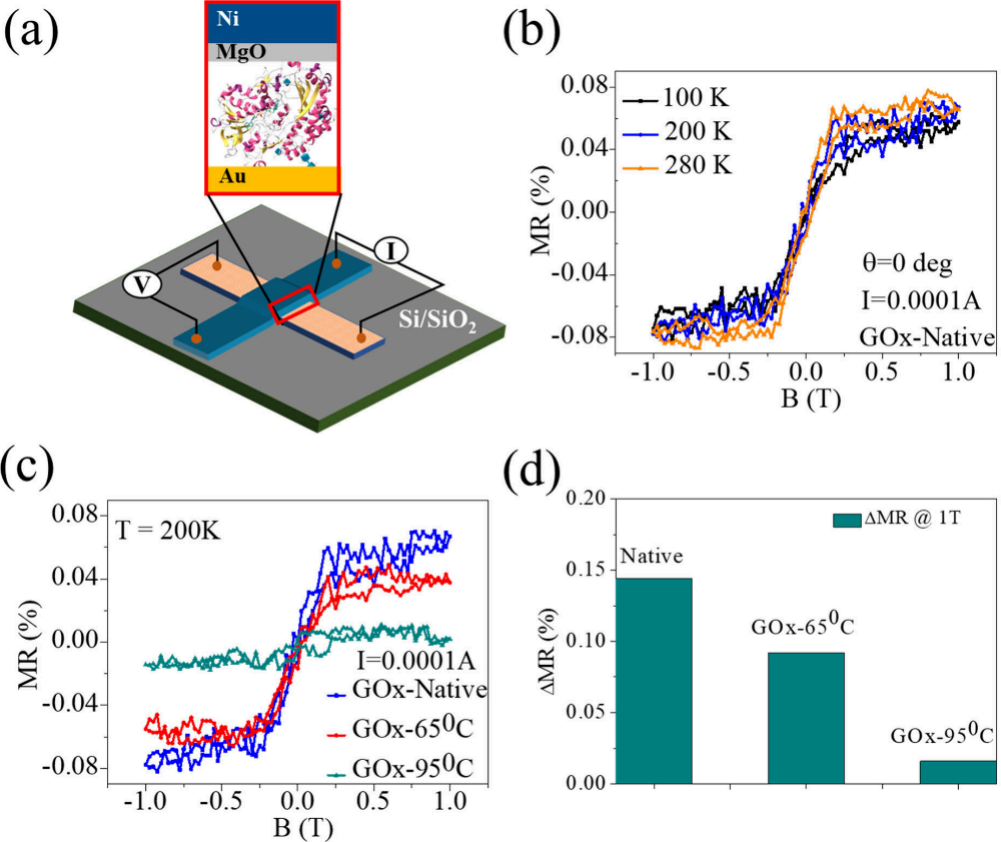
Magnetoresistance (MR) studies. (a) A scheme of the crossbar geometry spin-valve device for magnetoresistance measurements. The active area at the interface is shown in the zoomed image. The bottom electrode is made of gold (Au) on which the self-assemble monolayer (SAM) of GOx was adsorbed. A barrier layer of 1.5 nm MgO on the SAM followed by 60 nm ferromagnetic nickel (Ni) as top electrode. (b) The magnetic field-dependent MR at different measuring temperatures of native GOx. (c) MR signal as a function of magnetic field of native GOx and GOx denaturated at 65 and 95 °C. (d) The ΔMR(%) calculated at magnetic field of ±1T, where ΔMR (%) = |MR (%)|_–1T_ + |MR (%)|_+1T_ for native and denatured states of GOx. All the MR studies were measured at a constant current of 0.1 mAmp.

Spin polarization arising from the CISS effect is not a static property but a dynamic process driven by the interaction between the spin of a moving electron and the chiral potential of molecules. The efficiency of this spin-selective transport may be influenced by several factors, such as the molecular conformation, the electronic environment, and the nature of the underlying metal substrate. Here we demonstrate the importance of the protein’s secondary structure. The results indicate that spin polarization is mainly caused by the secondary structure and not the primary one. It is interesting that although the Hall studies were performed in solution, while the MR studies were conducted in a solid-state device, the results in both cases are consistent.

In summary, our results provide compelling evidence that the structural conformation of proteins plays a pivotal role in governing spin-selective electron transport via the CISS effect. Through a combined analysis of Hall-effect and magnetoresistance (MR) measurements, we demonstrate that native proteins, with well-preserved secondary structure, exhibit significantly enhanced spin polarization and asymmetric MR responses, even when the protein is located within a solid-state device. In contrast, denaturation, induced by elevated temperatures, leads to a disruption of the protein’s helical architecture, resulting in reduced spin filtering capabilities and notable reduction in both Hall-voltage slope and MR asymmetry. This study also indicates that biosystems, like proteins, retain their spin polarization properties even when imbedded within solid-state devices and therefore can serve in spintronic applications.^29^

## Supplementary Material





## Notes

Preparation and characterization of monolayers, fabrication and experimental set up of Hall experiments, magnetoresistance device fabrication, and experimental details (PDF)
Transparent Peer Review report available (PDF)
