# Supplementary material for: Protein Conformation Governs Spin-Selective Electron Transmission
Source: J Phys Chem Lett. 2025 Jun 17;16(25):6442–6. doi: 10.1021/acs.jpclett.5c01495 (PMC12207659; doi:10.1021/acs.jpclett.5c01495)
Supplement: Supplementary file 1 [file jz5c01495_si_001.pdf]

Supplementary Information

Protein Conformation Governs Spin-Selective Electron Transmission

Naupada Preeyanka, Tapan Kumar Das, Ron Naaman*

Department of Chemical and Biological Physics, Weizmann Institute of Science, Rehovot 7610001, Israel

Corresponding author email: ron.naaman@weizmann.ac.il

1) *Preparation and Characterization of Monolayers*

a) *Preparation:* The enzyme used exhibits a molecular weight of approximately 160 kDa and contains ≥65% protein by weight. Its specific activity ranges from 100,000 to 250,000 units per gram of solid, assuming no added oxygen. The product may contain trace amounts of other enzymes, including amylase, maltase, glycogenase, invertase, and galactose oxidase. The reproducibility of the results for different batches of the enzyme, indicates that there is no significant variation in the samples' purity.

The solid substrate, on which the protein was placed, was dipped inside 0.1 mM cystamine dihydrochloride solution (linker) for 12-14 hours. After the chemical adsorption of the linker, substrates were rinsed with ultrapure water (~4 times) to disgard unbound/excess cystamine dihydrochloride molecules, which were subjected to dry under nitrogen flow. Immediately, after that the substrates were again incubated with 0.19 mg/mL GOx solution, which was prepared in 0.1 M buffer at pH 8.0, for 12 hours. Now the substrates were washed the same buffer solution and then with ultrapure water to remove excess of GOx on the surface and dried under nitrogen flow.

b) *Characterization:* The monolayers were characterized using Circular Dichroism and Polarization modulation-infrared reflection-absorption spectroscopy.

Circular Dichroism (CD): The CD spectroscopy measurements were performed using a Chirascan spectrometer with a thermoelectrically controlled single cell holder. The

measurements were performed at 1 second per point, a 1 nm step size and with a bandwidth of 1 nm and enabling adaptive sampling for error points.

Polarization modulation-infrared reflection-absorption spectroscopy (PMIRRAS): PM-IRRAS was performed to characterize the linker (cystamine) and GOx attachment. We used Nicolet 6700 FTIR with PEM-90 photoelastic (Hinds Instruments, Hillsboro, OR) for our measurement. A 100 nm gold metal was deposited on the silicon substrate and monolayer of the linker as well as the GOx molecules were formed using the reported procedure. The Brewster angle of incidence was measured at 80 degree with 2000 scans.

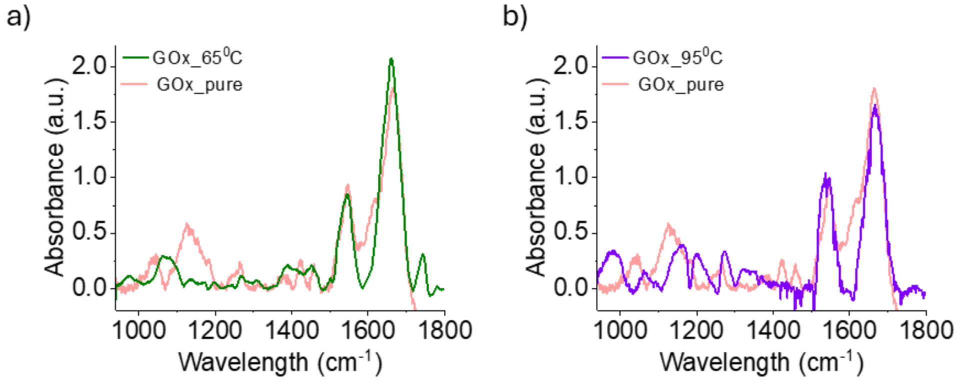

Figure S1: a) PMIRRAS of native GOx and its denatured state at 65^0C b) PMIRRAS of native GOx and its denatured state at 95^0C.

2) *Fabrication and Experimental Set up for Hall Voltage measurements*

   a) *Fabrication of Hall devices:* The Hall devices were fabricated in a class 1000 clean room by standard photolithography technique using AlGaN/GaN wafer on a sapphire substrate from NTT advanced technology corporation company. This wafer comprises of a nucleation layer, with an 1800 nm thick intrinsic-GaN layer. Above this intrinsic GaN layer, a 2DEG layer was encrypted upon which a 20 nm thick intrinsic AlGaN layer was embedded. A capping layer of 2 nm (GaN) was on top of it. To achieve ohmic contacts, metallic multilayer comprising of Ti – 20 nm, Al – 100 nm, Ni – 40 nm, Au – 40 nm, was annealed at 850^0C. The channel size with active area 500 μm in length and 40 μm in width was coated with 5 nm Au for the purpose of molecular absorption. Then these devices are subjected to self-assembled monolayer preparation (discussed later).

Once the monolayers grow, the devices were attached to the device holder using a double-sided tape, and the pads of the electrodes were connected to the chip holder using Au wire (through wire bonding). After checking the connections (avoid short circuiting), the contact was glued using high-quality RTV silicone glue. As the Hall measurements were performed in solution medium, a cell made up of PDMS (polydimethylsiloxane) gel was glued to the device which has a capacity of 200 µL. A thin glass coverslip coated with gold (120 nm) was used as a gate electrode which provides electric field for the polarization of the molecule. The whole set up was placed inside the Faraday cage during the measurements.

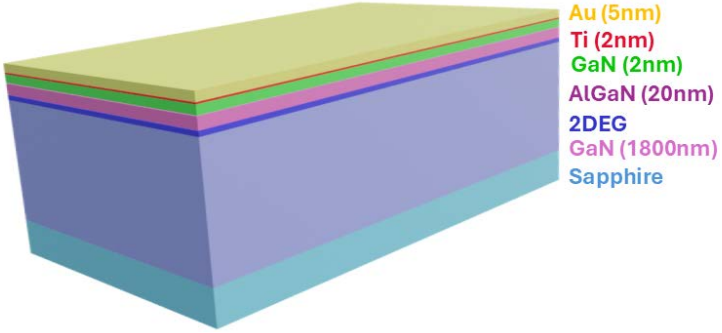

Figure S2. Schematic representation of the Hall device channel composition. The thin layer of Au promotes the adsorption of linker and the GOx molecules.

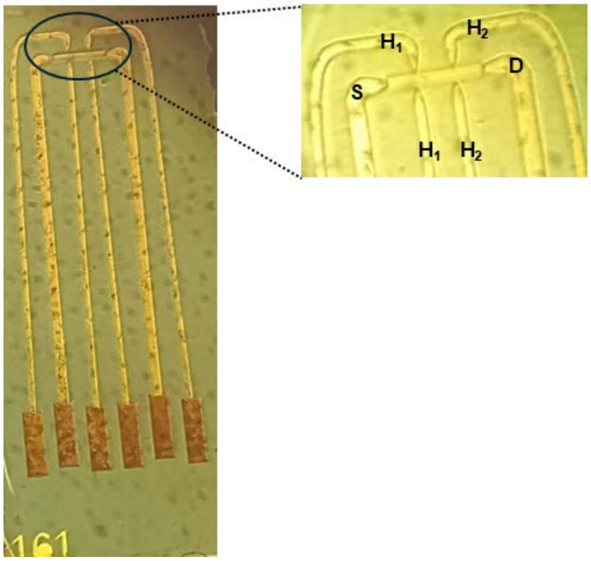

Figure S3: The microscopic image (optical) of the Hall device with the magnified channel showing source (S), drain (D) and Hall probes (H).

b) *Experimental Details:* A Keithley 2636A (dual channel) source was used to apply a constant current between the source (S) and the drain (D) electrodes with gate voltages varied according to the experimental requirements. These polarization measurements were measured using 0.1M phosphate (PBS) buffer medium. A Keithley 2182A nanovoltmeter was used to measure the Hall voltages (V_H). The voltage obtained due to the asymmetry of the Hall device was calibrated by maintaining the current in both forward and backward directions. The details of its calculation has been already reported elsewhere.[12]

Magnetoresistance device fabrication and measurement:

The magnetoresistance (MR) measurement was performed using spin-valve device configuration in a crossbar layout on SiO_2 substrate. The fabrication process began with the formation of the bottom electrode: a 2 μm wide conductive strip consisting of an 8nm titanium (Ti) adhesion layer topped with a 40nm gold (Au) layer, both deposited via optical lithography. The chiral self-assembled monolayer (SAM) of protein was grown on the bottom electrode. A 1.5 nm buffer layer of magnesium oxide (MgO) was then deposited via electron-beam evaporation to mitigate pinhole defects. Subsequently, the top electrode comprising 40 nm of ferromagnetic nickel (Ni) and an additional 20 nm of gold was thermally evaporated onto the MgO layer using a shadow mask with a 50 μm line width. All electrical and magnetic characterization were conducted in a closed-cycle cryogenic system from Cryogenic Ltd. Magnetoresistance was measured using a standard four-probe configuration under an applied magnetic field of up to ±1 T, oriented at various angles relative to the device plane. A constant current of 1 mA was supplied using a Keithley 2400 current source, while the corresponding voltage drop across the junction was recorded using a Keithley 2182A nanovoltmeter.
