# Supplementary material for: Protein Conformation Governs Spin-Selective Electron Transmission
Source: J Phys Chem Lett. 2025 Jun 17;16(25):6442–6. doi: 10.1021/acs.jpclett.5c01495 (PMC12207659; doi:10.1021/acs.jpclett.5c01495)
Supplement: Supplementary file 2 [file jz5c01495_si_002.pdf]

jz-2025-01495u.R1

Name: Peer Review Information for "Protein Conformation Governs Spin-Selective Electron Transmission"

First Round of Reviewer Comments

Reviewer: 1

Comments to the Author

Preeyanka et al. report the CISS effect in the native and denatured helical structure of flavin adenine dinucleotide. The authors utilized the Hall effect and solid-state MR devices to probe the spin-selectivity phenomena. They observed a reduction of the CISS effect upon elevated temperatures. This work highlights the importance of protein's secondary structure in efficient electron's spin transport. Considering the novelty of the manuscript, I recommend it for publication in JPC Lett. However, a minor revision is needed before the publication. Minor.

My specific points are as follows:

1. The CISS effect is highly affected by the purity of the compounds. The authors didn't mention the purity of the materials used for this study.

2. The length of the D-glucose oxidase should be given in Fig. 1a.

3. A color code will be helpful in Fig. 1 b.

4. Y-axix of Fig. 1d should be intensity, not absorption. The x-axis should be wavenumber (in cm-1). Please correct the same.

5. It will be nice to mention the amide II and amide I bands of PMIRRAS (Fig. 1d).

6. The value of MR is relatively less even at a high external magnetic field. The authors can comment on that.

7. There are many other proteins recently examined for CISS effect demonstration. For instance, doi.org/10.1063/5.0156479; 10.1016/j.chemphys.2022.111510. Please cite them in the Introduction section.

8. There should be a space between the numerical value and nm. For instance, 222nm should be 222 nm.

Reviewer: 2

Comments to the Author

Comments to the manuscript jz-2025-01495u "Protein conformation governs spin-selective electron transmission" by N. Preeyanka et al.

In this article the authors describe the effect of denaturation of a protein on the selectivity of the spin polarization of transmitted electrons. Proteins have both chiral carbon bonds as well as a chiral secondary structure. The authors show for glucose oxidase that the chiral secondary structure, where the atomic constituents assemble in a helical structure, is important for the development of a spin polarization of transmitted electrons. This is an important finding, and aligns with earlier observations that the helical structure of molecular entities is central for the spin selectivity. However, this importance of the helical secondary structure on the electron spin polarization had been shown earlier, even with one of the authors as co-author in these earlier papers. It was shown for oligo-DNA in the very first paper where spin polarization of electrons was observed (Science **2011**, *331*, 894) and also for oligo-peptides (J. Phys. Chem. C, **2015**, *119*, 14542), where denaturation of the molecules led to the disappearance of the spin polarization. In the latter one even the decrease of the amide I signal was observed, directly witnessing this denaturation, and being different from the present study. These references must be mentioned in a potentially revised version of the manuscript.

Therefore, when even on much smaller entities the secondary structure is important for the spin polarization, the inherent novelty in the present findings is marginal, and thus I do not recommend a publication in J. Phys. Chem. Letters, but rather in J. Phys. Chem. C.

Comments:

On page 2 it is stated that the correlation of the protein structure and the spin-dependent transport is monitored. I guess the protein structure is not monitored at all, but rather just a CD signal.

Further comments:

(i) The GOx molecules are bound via a linker to the gold surface, as stated on page 5. What is the influence of this linker (chiral or non-chiral ?) on the Hall signal ? Becomes it also denaturated ?

(ii) In fig. 2c values for the CD signal are missing. Given the inherent insensitivity and noise of CD signals, it is questionable, whether the difference between the curves are significant, in view of the general margin-of-error of such instruments.

(iii) Similar in fig. 1d: how large are the absorption values, and what is their margin-of-error ?

(iv) In fig. 2 it is not evident what part of the signal is called the Hall signal, the spikes or the height of the square wave function. It seems that only the height of the square wave function is taken, and not the spikes. Why not ? And please specify the signal.

(v) In the MR measurements the denatured proteins were not denatured in situ, but taken from a solution of denatured protein. Is the binding and order then still the same as for the intact molecules ? How is that proven ?

Typos:

Page 6, first paragraph, line 9: ..... injected spin is enhanced ....

Author's Response to Peer Review Comments:

Dear Editor,

Below please find our detailed response to the reviewers. Before addressing all the comments, we would like to elaborate on the issue of "novelty" as pointed by reviewer 2 and by your comments.

In response to this comment, we added now on page 3:

"It is important to note that, in the past, the dependence of the CISS effect's magnitude on secondary structure has primarily been studied in simple oligomers, such as double-stranded DNA and oligopeptides, where electron transmission occurs along essentially a single pathway. In proteins, however, their three-dimensional structure allows electrons to be conducted or displaced through multiple pathways. Consequently, one could imagine that the impact of denaturation on the CISS effect may not be as dramatic as in simpler systems. This study aims at verified the secondary structure effect on the magnitude of the CISS effect in a full-size protein."

We believe that this paragraph presents clearly the motivation and the novelty of this work. We have included the "TOC Graphic" after the abstract in the revised manuscript.

Reviewer: 1 Comments:

1. The CISS effect is highly affected by the purity of the compounds. The authors didn't mention the purity of the materials used for this study.

   Response: The enzyme exhibits a molecular weight of approximately 160 kDa and contains ≥65% protein by weight. Its specific activity ranges from 100,000 to 250,000 units per gram of solid, assuming no added oxygen. Regarding purity, the product may contain trace amounts of other enzymes, including amylase, maltase, glycogenase, invertase, and galactose oxidase. Hence, our study was conducted on a sample commonly used in biochemical studies. The reproducibility of the results for different batches of the enzyme, indicates that there is no significant variation in the samples' purity. A statement about the purity was added to the SI under "characterization".

2. The length of the D-glucose oxidase should be given in Fig. 1a.

Response: The length of D-glucose oxidase is ~8-10 nm (*J. Mater. Chem. B, 2023, 11, 2409–2416)*. We have now included the length of GOx in the revised manuscript.

3. A color code will be helpful in Fig. 1 b.
   Response: The color codes are amino acid residues of the FAD unit. It will require an additional figure if we have to mention all of the names of these amino acid residues. Since the exact structure of this enzyme is well documented and it is not essential for explaining our results, we decided not to provide this information in the manuscript.
4. Y-axix of Fig. 1d should be intensity, not absorption. The x-axis should be wavenumber (in cm-1). Please correct the same.
   Response: We have now corrected in the revised manuscript.
5. It will be nice to mention the amide II and amide I bands of PMIRRAS (Fig. 1d).
   Response: We have mentioned the amide II and amide I bands along with a short discussion (and references) in the revised manuscript page 5.
6. The value of MR is relatively less even at a high external magnetic field. The authors can comment on that.
   Response: The value of MR increases with the magnetic field till 0.25-0.3 Tesla, which is the nickel saturation magnetization. Hence, MR it is not expected to change, once the spin polarization in the metal is constant. It is reported in our previous work that, the MR values are relatively small, in this type of device, due to the presence of pinholes, which cause current leakage not through the chiral systems. Please see for example the discussion in JPC C 124, 1077610782 (2020).
7. There are many other proteins recently examined for CISS effect demonstration. For instance, doi.org/10.1063/5.0156479; 10.1016/j.chemphys.2022.111510. Please cite them in the Introduction section.
   Response: We have now included both references (as ref 5 and ref 6) in the revised manuscript.
8. There should be a space between the numerical value and nm. For instance, 222nm should be 222 nm.
   Response: We have corrected it in the revised manuscript.

<u>Reviewer 2</u>

In this article the authors describe the effect of denaturation of a protein on the selectivity of the spin polarization of transmitted electrons. Proteins have both chiral carbon bonds as well as a chiral secondary structure. The authors show for glucose oxidase that the chiral secondary structure, where the atomic constituents assemble in a helical structure, is important for the development of a spin polarization of transmitted electrons. This is an important finding, and aligns with earlier observations that the helical structure of molecular entities is central for the spin selectivity. However, this importance of the helical secondary structure on the electron spin polarization had

been shown earlier, even with one of the authors as co-author in these earlier papers. It was shown for oligo-DNA in the very first paper where spin polarization of electrons was observed (Science 2011, 331, 894) and also for oligo-peptides (J. Phys. Chem. C, 2015, 119, 14542), where denaturation of the molecules led to the disappearance of the spin polarization. In the latter one even the decrease of the amide I signal was observed, directly witnessing this denaturation, and being different from the present study. These references must be mentioned in a potentially revised version of the manuscript.

Therefore, when even on much smaller entities the secondary structure is important for the spin polarization, the inherent novelty in the present findings is marginal, and thus I do not recommend a publication in J. Phys. Chem. Letters, but rather in J. Phys. Chem. C.

Response: We now added a paragraph stating that:

"It is important to note that, in the past, the dependence of the CISS effect's magnitude on secondary structure has primarily been studied in simple oligomers, such as doublestranded DNA and oligopeptides, where electron transmission occurs along essentially a single pathway. In proteins, however, their three-dimensional structure allows electrons to be conducted or displaced through multiple pathways. Consequently, one could imagine that the impact of denaturation on the CISS effect may not be as dramatic as in simpler systems. This study aims at verified the secondary structure effect on the magnitude of the
CISS effect in a full-size protein."
We believe that this paragraph explains clearly the motivation and the novelty of this work.
Comments:

On page 2 it is stated that the correlation of the protein structure and the spin-dependent transport is monitored. I guess the protein structure is not monitored at all, but rather just a CD signal.

Response: The primary and secondary structure of protein both contribute to the chirality of protein, i.e. the existence of asymmetric carbon atoms and the helical configuration of the oligopeptide subunits. When we say correlation between the protein structure and spindependent transport, it means that from primary and secondary, it is mostly the secondary structure (basically the α-helices and β-sheets) which are contributing to the spindependent transport behavior. Our goal is to focus on the spin transport property of the protein (i.e. GOx) in solution and solid-sate devices retaining their α-helices and β-sheets.
Regarding monitoring of protein, we have used CD as well as PMIRRAS measurements which provide structural information of GOx. From CD, we confirmed that chirality is retained even though there has been some changes in the secondary structural conformation. From PMIRRAS, we concluded that the amide backbone is partially retained even after unfolding/denaturation.

Further comments:

(i) The GOx molecules are bound via a linker to the gold surface, as stated on page 5. What is the influence of this linker (chiral or non-chiral ?) on the Hall signal ? Becomes it also denaturated?

Response: The linker, cystamine dihydrochloride, is a non-chiral molecule whose role is to just bind the GOx to the gold surface. The linker structure is not affected at the temperatures applied in the experiment. We have checked the Hall signals with just attaching the linker on the hall device, but the signals were equal to that of the blank hall devices. Namely the linker by itself does not affect the Hall signal (see Figure below).

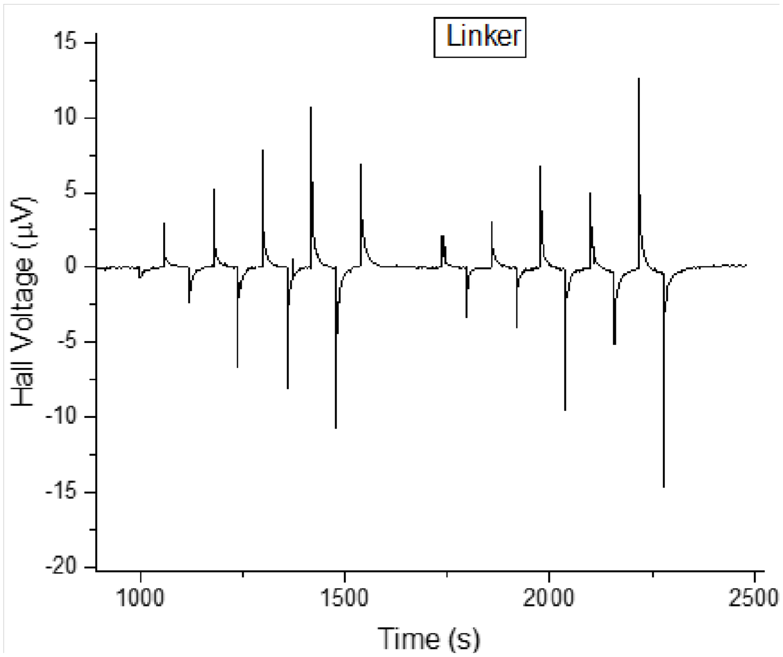

(ii) In fig. 2c values for the CD signal are missing. Given the inherent insensitivity and noise of CD signals, it is questionable, whether the difference between the curves are significant, in view of the general margin-of-error of such instruments.

Response: We have included the values in the revised manuscript.

These CD signals were normalized after subtracting the background. While doing the measurements, we have reduced the uncertainty by using the same cuvette for the background correction (filled with buffer).

Just to make things clear, we are not focused on the magnitude or how much is the difference in CD signal between the native and denatured state, rather our purpose is to highlight the change in shape of the curves, which is an indication of conformational changes in the active site of GOx. Below please see the separated graphs with the values that present clearly the denaturation effect.

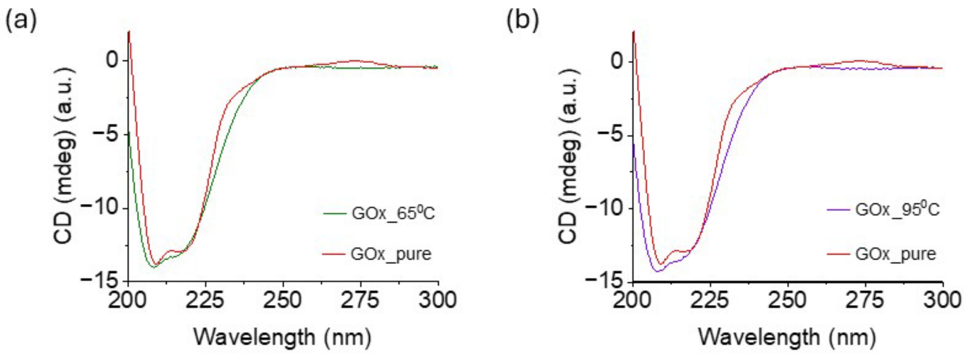

Figure: a) The CD signal for GOx in native and its denatured state at 65[1]C. b) The CD signal for GOx in native and its denatured state at 95^0C.

(iii) Similar in fig. 1d: how large are the absorption values, and what is their marginof-error ?

Response: We have separately plotted the IR absorption spectra, for native and of two of the denatured states. In the same way as the CD spectra, the values are baseline corrected. We are focused on the presence or absence of the peak and/or the change in the peak shape. As already discussed, the peak at 1420 cm^{-1} decreases at 65^0C and completely disappears at 95^0C, suggesting the FAD dissociation from protein or disruption of coupling between FAD and the tryptophan residue. The amide I band (shoulder of the peak at 1650cm^{-1}) disappears due to the denaturation, suggesting structural changes, while the amide II band (1540cm^{-1}) remains intact. Now, we have incorporated this information in the supporting information and also see below.

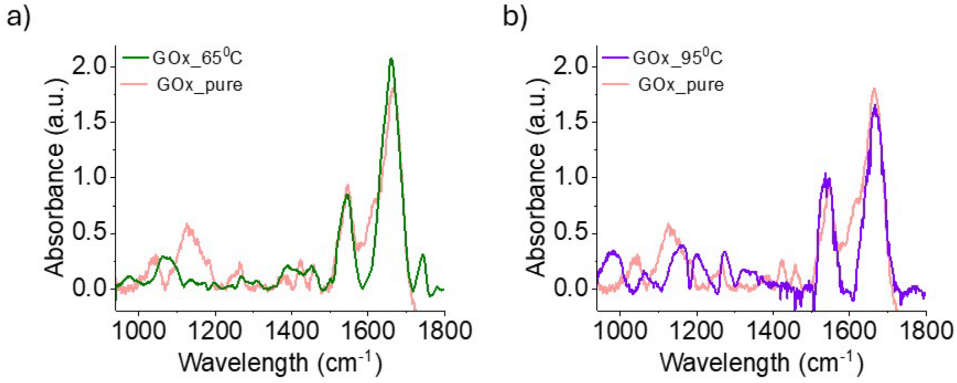

---

[1] C b) Figure S1: a) PMIRRAS of native GOx and its denatured state at 65 PMIRRAS of native GOx and its denatured state at 95^0C.

(iv) In fig. 2 it is not evident what part of the signal is called the Hall signal, the spikes or the height of the square wave function. It seems that only the height of the square wave function is taken, and not the spikes. Why not ? And please specify the signal.

Response: The explanation of the analysis of the Hall signal, are explained in details in the supporting information of our (ChemPhysChem. 2024, 25, e202400033) for GOx molecules. We now refer to this paper in the manuscript. The spikes of the Hall signal are not considered for calculating of the Hall signal since they result simply by switching the voltage on/off as shown in the response to comment (i). Since the spikes are simple a result of the electrical noise they are subtracted when calculating the Hall potential. This is now explained in the Figure caption of Figure 2.

(v) In the MR measurements the denatured proteins were not denatured in situ, but taken from a solution of denatured protein. Is the binding and order then still the same as for the intact molecules? How is that proven ?

Response: Throughout our studies (both Hall and MR), we have used solution of denatured protein to prepare the SAM. Hence the denaturation occurred in the same environment for the Hall and MR studies. To check the binding we made a SAM of native and denaturated protein on Au surface and confirmed the structure from the PMIRRAS measuremens.

Typos:

Page 6, first paragraph, line 9: ….. injected spin is enhanced ….

Response: We have corrected the typo.
